# Feasibility and impact of pharmacist-led penicillin allergy delabelling using the PEN-FAST scoring tool in a Canadian tertiary care hospital

**DOI:** 10.1017/ash.2026.10297

**Published:** 2026-02-16

**Authors:** Claire Kamaliddin, Amanda Driver, Rebecca Druken, Elissa Rennert-May, Irina Rajakumar

**Affiliations:** 1 Cummings School of Medicine, Department of Medicine, The University of Calgary, Calgary, AB, Canada; 2 Faculty of Pharmacy & Pharmaceutical Sciences, University of Alberta, Edmonton, AB, Canada; 3 Clinical Pharmacist, Foothills Medical Centre, Alberta Health Services, Calgary, AB, Canada; 4 Infectious Disease Physician and Antimicrobial Stewardship Site Lead, Department of Medicine, Section of Infectious Diseases, The University of Calgary, Calgary, AB, Canada; 5 Pharmacy Clinical Practice Leader, Alberta Health Services, Calgary, AB, Canada

## Abstract

**Background:**

Penicillins are the most frequently prescribed antibiotics for a broad range of infections. In North America, up to 15% of hospitalized patients report a penicillin allergy, but research has shown that 98% of these patients can tolerate penicillins. Removing inaccurate allergy labels is an essential component of antimicrobial stewardship. While allergy delabelling used to be complex or require an allergist referral, the emergence of new tools, such as the PEN-FAST score, facilitates direct delabeling of low-risk patients.

**Objectives:**

The primary objective of this study was to trial the use of the PEN-FAST scoring tool at a major tertiary care center in Canada. Secondary objectives included measuring the pharmacy workload associated with the delabeling process.

**Methods:**

A prospective pilot study was implemented at a Canadian tertiary care hospital to identify new patients with a penicillin allergy label, perform a review of their medical history, obtain a PEN-FAST score, and if applicable, implement an oral challenge with amoxicillin.

**Results:**

Most of the 155 screened patients were delabeled based on their medical history. Twenty-nine patients were eligible for an oral challenge, and three challenges were conducted.

**Conclusion:**

PEN-FAST scoring in combination with direct oral challenge is a practical tool that can be prospectively implemented by pharmacists.

## Introduction

In North America, 15% of the hospitalized population are labeled with a penicillin allergy.^1,2^ However, these labels are often incorrect, with patients reporting a distant and unclear history of minor side effects (such as a rash or gastrointestinal symptoms) or a coincidental event (headache, cutaneous eruption due to an underlying infection).^3^ To further support risk stratification at the point of care and support allergy delabeling initiatives, a fast and practical scoring system, “PEN-FAST,” was recently validated.^4–7^ PEN-FAST is a sequential five point questionnaire reporting a numerical score. A PEN-FAST score below three is considered low-risk and qualifies for a direct oral challenge.

The primary objective was to trial using PEN-FAST systematically at a major tertiary care center. Secondary objectives included measuring the impact on daily patient care workflow associated with the delabeling process.

## Methods

All adult patients admitted to Foothills Medical Centre (a major university hospital (1093 beds) in Calgary, Alberta, Canada) from January 29^th^ to February 16^th^, 2024, with a label of a penicillin allergy in the electronic medical record were identified by workbench reports and eligible. There was no standardized approach to allergy delabeling at the hospital. Exclusion criteria followed the PALACE trial.^8^ A comprehensive medication history review was conducted by the pharmacist via screening of the Electronic Medical Record (EMR) and outpatient prescription fills in the provincial pharmacy record system. If the described symptoms were consistent with an adverse effect, not immunologically mediated, or if the patient had subsequently received a penicillin without any adverse effect, the allergy label was removed directly. The remainder of the patients qualified for PEN-FAST scoring according to published protocols.^4,5^ Patients who scored less than 3 were offered an oral challenge with 250 mg amoxicillin as per prior routine clinical practice (including enhanced monitoring and an “as needed” order for IM epinephrine and IV diphenhydramine). Upon completing this process, the pharmacist assessed if the patient could be delabeled. If delabeled, the patient was educated regarding the removal of the penicillin allergy, the EMR was updated and their primary care provider and community pharmacy informed via eFax by standard letter.

This study was a quality improvement project exempted from Research Ethic Board (REB) approval by the University of Albertaand assessed through the ARECCI tool.

## Results

### Screening process

**Figure 1.**
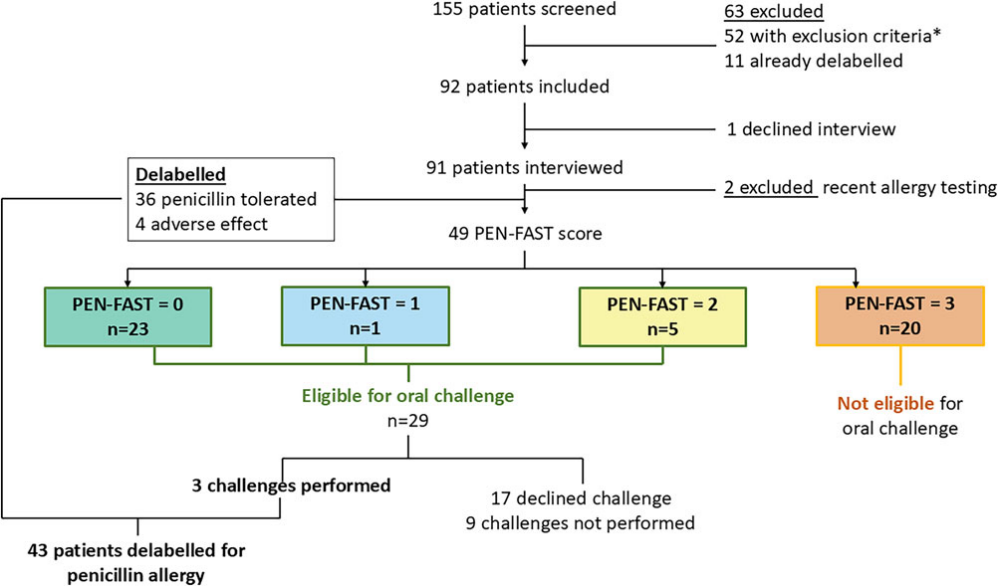
Patient flow and results of allergy assessment.

A median of 51 patients was on the daily EMR generated report (range 12–69), among which a median of 10 per day were new (range 3–37), leading to a total of 155 patients screened during the study period (Figure 1).

### Outcomes

Ninety-two patients were retained from the screening phase. One patient declined the interview (n = 1/92, 1.1%). Amongst the 91 remaining patients that were interviewed, two patients were excluded based on recent allergy testing, and forty (n = 40/91, 43.9%) were delabeled based on their history alone and, therefore, did not meet the criteria for an amoxicillin oral challenge (Figure 1). PEN-FAST scoring was performed on 49 patients (n = 23/49, 46.9% scored 0, n = 1/49, 2.1% scored 1, n = 5/49, 10.2% scored 2, and n = 20/49, 40.8% scored 3). Twenty-nine patients (PEN-FAST < 3) qualified for an oral challenge, of which most declined the challenge (n = 17/29, 58.6%) due to timing or clinical status. Amongst the twelve patients who were agreeable to the challenge, three challenges were ordered and performed, and all patients tolerated the challenge with no reaction and were subsequently delabeled (Figure 1). In total, 43 (n = 43/92; 46.7%) patients were delabeled for penicillin allergy through this intervention (Supplementary material).

### Pharmacist workload

A total of 155 screenings occurred over 15 working days. The average daily workload associated with the screening, interview, and delabeling process was 315 min (SD: 75 min, range 230 min–530 min), corresponding to 19.7 min per patient. The average daily screening duration was 133 min (SD: 37 min, an average of 8.3 min per patient), leading to an average of five fully conducted interviews (SD: 2). The daily workload associated with conducting the interviews was 72 min, or 14.4 min/interview (SD: 27 min). While communicating with the care team was not time-consuming (average 6 min per day, SD: 9 min), documentation and reporting time was significant, with an average of 98 min per day (SD: 42 min).

## Discussion

This project was the pilot implementation of a formalized penicillin allergy delabeling program at a university hospital in Calgary, Alberta, Canada. Overall, the program showed the feasibility of direct delabeling based on medical records review, with 43.5% of eligible patients being delabeled directly. Amongst the patients that were scored using the PEN-FAST tool, the majority (59%) scored ≤2, showing that only a limited number of patients require a referral to allergy specialist services. These results are consistent with the literature, as allergies may wane over time, and the risk of repeated IgE-mediated hypersensitivity reactions to structurally related antimicrobials diminishes by up to 80% over 10 years.^9^

The secondary objective of the pilot study was to measure the impact of the workflow in the care system. The observed pharmacist workload of 5 h 15 min to assess an average of 10 patients per day, shows that implementing PEN-FAST would take up a significant portion of a pharmacist’s workday. Strategies to enable this type of initiative include involving pharmacy trainees (students and residents) and deploying pharmacy technicians to conduct the allergy history component of the assessment.

In summary, this study showed that electronic medical records, particularly when including community pharmacy prescriptions, can easily be leveraged to support pharmacist-led penicillin allergy delabeling strategies.

## Supporting information

10.1017/ash.2026.10297.sm001 Kamaliddin et al. supplementary material 1 Kamaliddin et al. supplementary material

10.1017/ash.2026.10297.sm002 Kamaliddin et al. supplementary material 2 Kamaliddin et al. supplementary material

## Data Availability

Data are available from the authors upon reasonable request and with permission of Alberta Health Services.

## Supplementary material

The supplementary material for this article can be found at https://doi.org/10.1017/ash.2026.10297.
